# High salt diet alleviates disease severity in native experimental autoimmune uveitis

**DOI:** 10.3389/fopht.2024.1370374

**Published:** 2024-05-31

**Authors:** Naomi Derluyn, Vincent Foucart, Marko Verce, Rami Abdo, Louis Vaudoisey, Deborah Lipski, Véronique Flamand, Amandine Everard, Catherine Bruyns, François Willermain

**Affiliations:** ^1^ Institute of Interdisciplinary Research (IRIBHM), Université Libre de Bruxelles (ULB), Brussels, Belgium; ^2^ Department of Ophthalmology, CHU Saint-Pierre, Université Libre de Bruxelles (ULB), Brussels, Belgium; ^3^ Department of Ophthalmology, CHU Brugmann, Université Libre de Bruxelles (ULB), Brussels, Belgium; ^4^ Metabolism and Nutrition Research Group, Louvain Drug Research Institute, UCLouvain, Université Catholique de Louvain, Brussels, Belgium; ^5^ WELBIO Department, WEL Research Institute, Walloon Excellence in Life Sciences and BIOtechnology (WELBIO), Wavre, Belgium; ^6^ Department of Ophthalmology, Hôpital Universitaire de Bruxelles (HUB) - Hôpital Erasme, Université Libre de Bruxelles (ULB), Brussels, Belgium; ^7^ Institute for Medical Immunology, Université Libre de Bruxelles (ULB), Charleroi, Belgium

**Keywords:** autoimmunity, inflammation, uveitis, EAU, high salt diet, gut microbiome

## Abstract

**Background:**

Recent studies reported a link between high salt diet (HSD) and clinical exacerbation in mouse models of autoimmune diseases, mainly through the induction of pathogenic Th17 cells and/or HSD-induced dysbiosis. However, the topic remains controversial and not fully understood.

**Purpose:**

In this study, we investigated the effects of HSD on the development of experimental autoimmune uveitis (EAU) in C57BL/6J mice.

**Methods and results:**

Unexpectedly, our data showed a significant attenuating effect of HSD on disease severity of native EAU, induced by direct immunization with IRBP peptide. That said, HSD had no effect on EAU disease severity induced by adoptive transfer of semi-purified auto-reactive IRBP-specific T lymphocytes. Accordingly, HSD did not affect IRBP-specific systemic afferent immune response as attested by no HSD-linked changes in T lymphocytes proliferation, cytokine production and Treg proportion. Gut microbiota analysis from cecal samples in naïve and EAU mice demonstrated that HSD affected differentially α-diversity between groups, whereas β-diversity was significantly modified in all groups. Unknown *Tannerellaceae* was the only taxon associated to HSD exposure in all treatment groups. Interestingly, a significantly higher abundance of unknown *Gastranaerophilales*, with potential anti-inflammatory properties, appeared in HSD-fed native EAU mice, only.

**Discussion:**

In conclusion, our study suggests a possible impact of HSD on gut microbiota composition and consequently on development and clinical severity of EAU. Further studies are required to investigate the potential beneficial role of *Gastranaerophilales* in EAU.

## 1 Introduction

The term uveitis refers to potentially blinding inflammatory diseases of the eye that are responsible for 5-10% of the visually impaired worldwide. It is characterized by inflammation of the uvea, which is composed of the iris, the ciliary body, and the choroid. In addition to the uvea, uveitis can also involve the vitreous, the retina and the optic nerve (1). Uveitis is usually classified according to etiology, more specifically into infectious (caused by a pathogen) and non-infectious uveitis. Non-infectious uveitis can occur in its own isolated form but also as part of a systemic disease, in a context of auto-inflammatory or autoimmune disease. It is a relatively common disease in developed countries and affects mostly young adults (2).

The discovery of Interphotoreceptor Retinoid-Binding Protein (IRBP) as a highly uveitogenic antigen (Ag) in Lewis rats promoted fundamental research on autoimmune uveitis (3). The mouse model of experimental autoimmune uveitis (EAU), either native EAU (induced by direct immunization) or EAU by adoptive transfer (AT) of IRBP-specific activated T lymphocytes, is representative of posterior non-infectious uveitis in humans (4–6).

The systemic response triggered by immunization begins with its afferent phase, during which interferon (IFN)-γ-producing Th1-type and interleukin (IL)-17-producing Th17-type CD4^+^ T cells are activated by antigen-presenting cells (APCs) in the peripheral lymphoid organs (7). Then, during the efferent phase, these activated autoreactive T cells randomly migrate to the eye and recognize the specific Ag against which they are directed (8). The endothelial cells of the blood-retinal barrier (BRB) will, under the influence of cytokines and chemokines secreted in this inflammatory context, express adhesion molecules and reduce the expression of tight junction proteins. This causes a weakening of the BRB and leads to the recruitment of circulating leukocytes, resulting in deleterious effects on the retina (9).

The incidence of autoimmune diseases has increased substantially in western countries in recent years. Western diet, which is particularly rich in salt and already commonly identified as a major risk factor for cardiovascular disease, appears to be a potential risk factor for the development of autoimmune diseases (10). Sodium ion has been shown to promote a pro-inflammatory state by stimulating the differentiation of Th17 cells and by modulating regulatory T cells (Treg) and M1 and M2 macrophages (11). Kleinewietfeld et al. showed that high concentrations of NaCl *in vitro* increase the induction of highly pathogenic Th17 cells secreting more pro-inflammatory cytokines such as IL17A, GM-CSF, TNFɑ and IL-2 (12). *In vivo*, in a variety of autoimmune disease models including a mouse model of experimental autoimmune encephalomyelitis (EAE), the deleterious role of the hypersaline diet (HSD) in the development and severity of these diseases has been proven (11–13).

The involvement of commensal gut microbiota in the pathogenesis and modulation of autoimmune diseases, including autoimmune uveitis, appears well established. Gut microbiota can act as a trigger for the activation of autoreactive T cells in the intestinal lamina propria through antigenic mimicry and microbial adjuvant effects. A role for microbial metabolites in modulating uveitis disease has also been reported (14, 15). One of the effects of HSD is actually to affect the gut microbiota by modulating the relative abundance of specific microorganisms, in particular by reducing the abundance of *Lactobacillus spp*, thereby inducing highly pathogenic Th17 cells (11, 16).

The precise mechanisms of the interaction between the pathophysiological pathways of autoimmune uveitis, the gut microbiota, the immune system and environmental factors remain to be elucidated. A study of the effect of HSD on the expression and development of autoimmune uveitis and a better understanding of the associated underlying mechanisms may provide a new approach to the management of autoimmune diseases.

The aim of this study was to investigate the mechanisms by which HSD affects the retina and interacts with the development of EAU in mice. Different factors were studied comparatively between diseased and healthy mice subjected to HSD versus control diet, such as the severity of the disease, its link with the antigen-specific immune response during the induction phase of the disease and at a stage of established EAU, the BRB integrity and structure in the retina and the gut microbiome.

## 2 Material and methods

### 2.1 Mice and diet regimen

Pathogen-free female C57BL/6J mice (6 weeks old), purchased from Janvier (Genest St Isle, France) were housed under specific pathogen-free conditions at the animal facility of the Université Libre de Bruxelles, Belgium in accordance with European guidelines. Ethics approval (protocol 669N) was obtained from the local Ethics Committee (Comission d’Ethique du Bien-Être Animal, CEBEA, Faculté de Médecine, Université Libre de Bruxelles).

Two groups of C57BL/6J mice were fed during 4 weeks with either a control diet (Sniff EF R/M control E15430-04) and water *ad libitum*, or a high salt diet (HSD) (Sniff EF R/M high salt, NaCl 4%, E15431-34) and salted water 1% *ad libitum*. Diets were irradiated before use.

### 2.2 Induction of experimental autoimmune uveitis

C57BL/6J mice were immunized for EAU by subcutaneous injections into each thigh with 50 µL of a solution containing 500µg/100µL interphotoreceptor retinoid-binding protein (IRBP) peptide emulsified 1:1 in complete Freund’s adjuvant (CFA), supplemented with 2.5 mg/mL inactivated *Mycobacterium tuberculosis*. All animals received simultaneously an additional 10 µL intraperitoneal injection of 1 μg of *Bordetella pertussis* toxin (PTX). IRBP peptide (GPTHLFQPSLVLDMAKVLD), representing a sequence of the human IRBP, was synthesized by New England Peptide (Gardner, MA., USA). PTX and CFA were purchased from Sigma-Aldrich (Bornem, Belgium).

EAU by adoptive cell transfer (AT) of autoreactive lymphocytes was induced following the protocol of Shao H et al. (17). Briefly, animals were immunized as mentioned above. Twelve days after immunization, mice were sacrificed by cervical dislocation and their spleen and draining lymph nodes dissected and dissociated. Spleen cell suspensions were enriched in T lymphocytes through passage on nylon wool fiber columns, then pooled with total lymph node cells and restimulated *in vitro* with IRBP peptide (1μg/ml). After 2 days in culture, cells were injected intraperitoneally into naïve C57BL/6J mice (3×10^6^ cells/mouse) previously subjected for 4 weeks to an identical diet, respectively.

Disease induction was repeated at several months interval, three times for native EAU, and twice for EAU by AT, involving 5 to 12 mice per group each time.

### 2.3 Disease grading

A clinical grading was performed 21 days after induction of the disease. Mice were anesthetized by gas. Pupils were dilated with tropicamid (5 mg/ml) and phenylephrine (1.5 mg/ml) and fundoscopic examination was done under a surgical microscope (Zeiss, Goüttingen, Germany) by using a cover slip coated in a viscoelastic gel (synthetic polymer of acrylic acid 2 mg/g, Vidisic, Tramedico, Belgium) and positioned on the cornea. The double-blind clinical grading was performed independently by two ophthalmologists, based on a system adapted from Xu et al. (18). Briefly, vitritis, optic neuritis, retinitis and vasculitis were separately scored in each eye, from 0 (no disease) to 4 (highly severe disease) with half point increments and averaged to generate the clinical score of the eye on a scale from 1 to 4. The clinical score attributed to one mouse corresponds to the mean of the scores of the 2 eyes. Some mice developed cataract, independently of the diet.

### 2.4 Characterization of IRBP peptide-specific T cells

Twelve days after immunization (induction phase of the disease) or twenty-one days after immunization (established disease) mice subjected to control or HSD were sacrificed and their spleen and draining lymph nodes dissected and dissociated. Spleen cell suspensions were enriched in T lymphocytes through passage on nylon wool fiber columns, then pooled with total lymph node cells and 5x10^6^ cells/2ml/well were re-stimulated *in vitro* during 2 days with or without IRBP peptide (1 μg/ml).

In some experiments, we also investigated the effect of adding NaCl during *in vitro* culture in mice not subjected to a specific diet. They similarly were killed on day 12 after immunization and their splenic and lymph node T cells were restimulated *in vitro* for 72h at 5x10^6^ cells/2mL/well in LT medium alone or supplemented with IRBP peptide (1µg/ml) or with Dynabeads Mouse T-activator CD3/CD28 (5µL/well), with or without addition of NaCl 40mM in the culture wells.

For cell proliferation analysis, an EdU test (Click-iT™ Plus EdU Flow Cytometry Assay Kits from Invitrogen, ThermoFisher™ Scientific, Merelbeke, Belgium) was used following protocol provided by the manufacturer. Cell proliferation was quantified on single cell suspensions 48-72h later, adding EdU 10µM for the final 18 hours. CD3+CD4+ T cells were identified by flow cytometry (LSR-Fortessa flow cytometer using CellQuest Software (BD Biosciences) based on membrane staining with PE Hamster anti-mouse CD3 (BD Pharmigen) and PerCP-Cy5.5 Rat anti-mouse CD4 (BD Pharmigen). Proliferating cells were identified by labelling with Alexa Fluor 488 picolyl azide. Fluorescence minus one (FMO) controls were used for accurate gating.

For secreted cytokine assays, supernatants of the same wells were collected and frozen at -20°C. Cytokine secretion of IFNγ, IL-17A and GM-CSF was quantified using specific ELISA tests as part of a collaboration with Prof V. Flamand (IMI Gosselies).

For intracellular cytokine assays, the cultured cells were stimulated for a final 4h with PMA (50 ng/mL) and ionomycin (1 μg/mL) (Sigma-Aldrich) in the presence of BD Golgistop Protein Transport Inhibitor (1μL/mL) (BD Bioscience). IFNγ-producing CD4+ Th1 cells and IL-17A-producing CD4+ Th17 cells were detected by flow cytometry using the mouse Th1/Th17 Phenotyping Kit (BD Bioscience) according to the manufacturer’s instructions. In some assays, such detection was combined with Click-iT™ Plus EdU Flow Cytometry Assay Kits from Invitrogen (ThermoFisher™ Scientific) for EdU proliferation analysis.

The presence of Tregs was assessed by a multicolor flow cytometric analysis of FOXp3 expression within CD4+CD25+ splenocytes. Briefly, cells were first incubated with relevant surface antibodies (BV421 Rat Anti-Mouse CD4 and APC Rat Anti-Mouse CD25 (BD Biosciences)) before being fixed and permeabilized using the Transcription Factor Buffer Set (BD Biosciences), according to the manufacturer’s instructions. Next, intracellular expression of transcription factor 3 (FOXp3) was performed by staining with PE Mouse Anti-Mouse FOXp3 (BD Biosciences). Relevant control isotype (PE Mouse IgG1, κ Isotype Control, BD Biosciences) was used as fluorescence minus one (FMO) control. Samples were analyzed on a BD LSR Fortessa-x20 using FACSDiva (BD Cytometry Systems).

### 2.5 Immunohistology

Eyes were collected 21 days after disease induction and immediately fixed in 4% buffered formaldehyde, paraffin-embedded, and sectioned (3µm thick) at different levels, including in the proximity of the optic nerve. Eye sections were stained with hematoxylin and eosin (Sigma-Aldrich).

For immunofluorescence stainings, eye sections were first incubated overnight at 4°C with a mouse monoclonal to glial fibrillary acidic protein (GFAP; dilution 1:400; Millipore Sigma-Aldrich, Billerica, MA) as primary antibody. The sections were then incubated with donkey anti-mouse IgG coupled to cyanin3 (red fluorochrome; dilution 1:300; Jackson Immunoresearch). Cell nuclei were identified by Hoechst staining (1:1000; Sigma-Aldrich). Finally, sections were mounted using ProLong^™^Gold Antifade Mountant (Thermo Fisher Scientific). Negative control sections were incubated either with secondary antibody or with primary antibody alone. Immunofluorescent pictures were acquired using AxioImager Z1 equipped with an AxioCamMRm camera (Zeiss) using the z-stack mode of the Axiovision acquisition software.

### 2.6 Microbiome analysis of mouse cecal samples

This analysis was conducted in collaboration with UCLouvain, Université catholique de Louvain, Louvain Drug Research Institute, Metabolism and Nutrition Research Group, Walloon Excellence in Life Sciences and BIOtechnology (WELBIO), Brussels, Belgium.

Briefly, cecal contents were collected and kept frozen at −80°C until use. Metagenomic DNA was extracted from the cecal content using a QIAamp DNA Stool Mini Kit (Qiagen, Hilden, Germany) according to the manufacturer’s instructions with modifications. The V3–V4 region of the 16S rRNA gene was amplified from the cecal microbiota of the mice using the following primers: rambacV3F (5’-CCTACGGGAGGCAGCAG-3’) and rambacV4R (5’-GGACTACHVGGGTWTCTAAT-3’). Purified amplicons were sequenced utilizing an Illumina MiSeq following the manufacturer’s guidelines. Sequencing was performed at MR DNA (www.mrdnalab.com, Shallowater, TX, USA). Sequence reads were demultiplexed and processed using the QIIME2 pipeline (q2cli 2021.4.0) (19), including primer removal using cutadapt (20) and denoising with DADA2 (21), modifying the following DADA2 parameters: maximum expected error = 2, truncation length = 252 nt, minimum fold parent over abundance = 4. To ensure quality, only forward sequencing reads were used. For the 58 samples analyzed, 4849 ASVs have been identified, which were then decontaminated by mapping to the mouse genome GRCm39 (GCF_000001635.27), resulting in 4847 decontaminated ASVs. The taxonomic assignment of ASVs was performed using a classifier based on the SILVA 138.1 SSURef NR99 database (22) that was dereplicated and trimmed to the V3-V4 region using RESCRIPt (23). After this processing, samples contained between 16917 and 92670 sequence reads, with the median and mean number of sequence reads of 52860 and 53636, respectively. The taxonomic composition of samples was visualized using the collection of R packages “tidyverse” (24). The sequencing data were submitted to the European Nucleotide Archive (ENA/EBI) and are available under the study accession number PRJEB65938.

### 2.7 Statistical analysis

Mann-Whitney U-test was used to assess the effect of HSD on disease severity, proliferation of T lymphocytes and their cytokine secretion. Wilcoxon test was used to assess the effect of NaCl *in vitro* on proliferation of T cells and their cytokine secretion. These statistical analyses were done using GraphPad Prism (Graphpad Software Inc., La Jolla, CA).

For the gut microbiota analysis, the statistical analysis was performed by UCLouvain, Université Catholique de Louvain, Louvain Drug Research Institute, Metabolism and Nutrition Research Group, Walloon Excellence in Life Sciences and BIOtechnology (WELBIO), Brussels, Belgium. Diversity metrics were calculated using a sampling depth of 30500 reads. Two samples were excluded from statistical analyses due to a comparatively low number of sequence reads. PCoA was performed using the weighted and unweighted UniFrac distances with QIIME2. The PCoA plots were visualized using the “tidyverse” collection of R packages “tidyverse” (24). Differences in alpha-diversity metrics of gut microbiota samples were assessed in QIIME2 using the Kruskal-Wallis test, and differences in beta-diversity of gut microbiota samples were assessed in QIIME2 using PERMANOVA (25) and PERMDISP, using 9999 permutations. To assess whether any ASVs or genera were more abundant in any of the groups, a compositional data analysis was performed by applying ALDEx2 (26) on filtered ASV/genus tables, wherein ASVs/genera represented in at least 4 samples and by more than 50 sequence reads in total were retained.

## 3 Results

**Figure 1:**
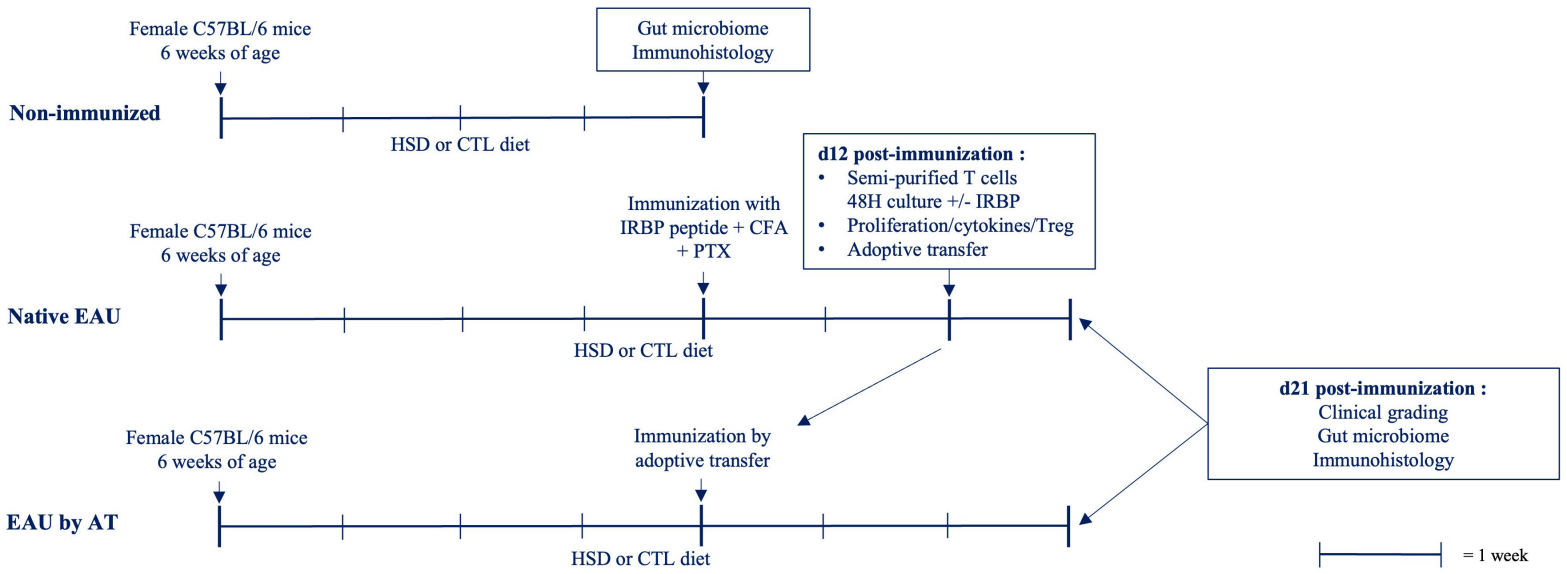
Experimental schedule. C57BL/6 mice, housed in a SPF environment, were submitted to HSD or control diet for 4 weeks. Native EAU was induced by s.c. injection of IRBP peptide emulsified in CFA and a simultaneous i.p. injection of PTX. For EAU by AT, spleen and draining lymph nodes from native EAU mice were collected 12 days after immunization. Semi-purified T cells were restimulated *in vitro* with IRBP peptide during 2 days before i.p. injection into naïve C57BL/6 mice previously submitted for 4 weeks to similar diets. Mice were kept on the same respective diets during disease induction. Different parameters were examined comparatively between HSD versus control diet. The clinical grade of EAU was determined on day 21. The antigen-specific response was assessed during the induction phase of the disease (d12) by testing the proliferation rate and the cytokine production of T cells in response to IRBP as well as the proportion of Treg. Immunohistology staining was performed on paraffin-embedded eye sections of healthy and diseased mice. An analysis of the intestinal microbiome on cecal samples was done on healthy and diseased mice submitted to both diets.

The aim of this study was to test whether high salt diet (HSD) would influence the development and severity of experimental autoimmune uveitis (EAU) in C57BL/6 mice. The whole experimental procedure we followed is illustrated in **Figure 1**. Briefly, 6-weeks old C57BL/6 mice, housed in a SPF environment, were submitted to HSD or control (CTL) diet for 4 weeks. Native EAU was then induced by subcutaneous (s.c.) injection of IRBP peptide emulsified in CFA and a simultaneous intraperitoneal (i.p.) injection of PTX. For EAU by adoptive transfer (AT), semi-purified T cells were restimulated *in vitro* with IRBP peptide during 2 days before i.p. injection into naïve C57BL/6 mice previously submitted for 4 weeks to similar diets. Mice were kept on the same respective diets during disease induction. Different parameters were examined comparatively between HSD and CTL diet. The clinical grade of EAU was determined on day 21 after immunization. The antigen-specific response was assessed during the induction phase of the disease (day 12 after immunization) by testing the proliferation rate and the cytokine production of T cells in response to IRBP as well as the presence of regulatory T cells (Treg). Immunohistology staining was performed on paraffin-embedded eye sections of healthy and diseased mice. An analysis of the intestinal microbiome on cecal samples was finally done on healthy and diseased mice submitted to both diets. All data were collected from individual mice.

### 3.1 *In vivo* assessment of EAU severity

**Figure 2:**
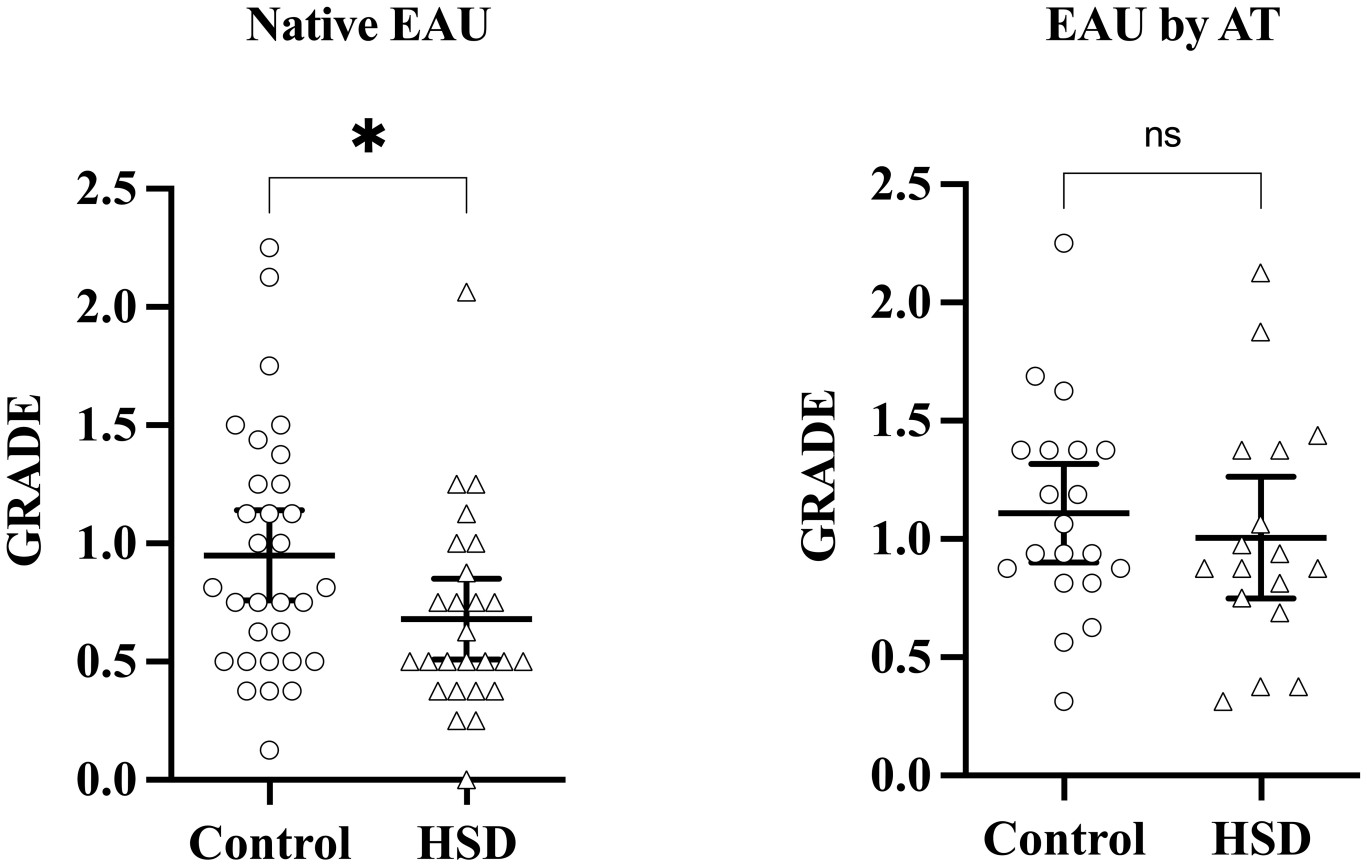
HSD attenuates uveitis development in the native EAU model. Disease induction was repeated at several months interval, three times for native EAU, and twice for EAU by AT, involving 5 to 12 mice per group each time. Disease severity was assessed on day 21 after induction, at the stage of established EAU, by performing clinical grading of vitritis, retinitis, optic neuritis and vasculitis. Each symbol represents the mean clinical grade of the 2 eyes of one individual mouse. Mann-Whitney test was used to compare disease severity between diets. Values are presented as means ± 95% CI. “ns” not significant; *p < 0,05.

Disease severity was assessed at the stage of established EAU, 21 days after disease induction by examination of eye fundus. Clinical grades are shown in **Figure 2**. A statistically significant attenuating effect of HSD on disease severity is observed in native EAU (p = 0,0288) whereas no difference is observed in EAU by AT (p = 0,4442).

### 3.2 *Ex vivo* characterization of IRBP-specific immune response in diseased mice during the induction phase of the disease (day 12)

**Figure 3:**
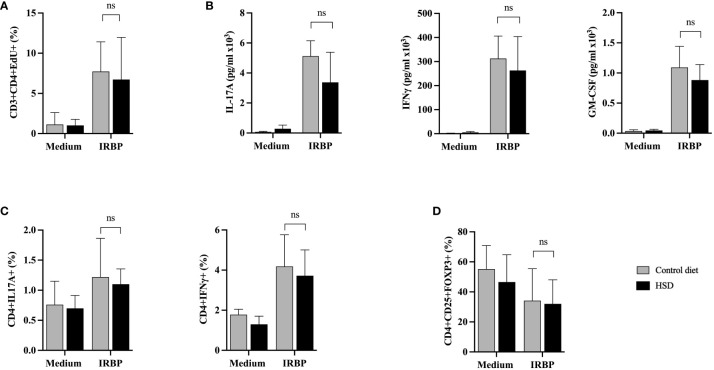
HSD does not affect ex vivo proliferation of IRBP-specific T cells nor cytokine production or Treg presence in diseased mice during the afferent phase. Twelve days after s.c. immunization with IRBP peptide, HSD and control diet individual mice were sacrificed and their spleen and draining lymph nodes collected. Semi-purified T cells were restimulated *in vitro* with or without IRBP peptide for 48 hours. **(A)** Cell proliferation was assessed by flow cytometry using an EdU test (CTL n = 9; HSD n = 8; 3 independent experiments). **(B)** IL-17A, IFNγ or GM-CSF cytokine secretion was assessed by specific ELISA on same cell culture supernatants (CTL n = 4; HSD n = 4; 2 independent experiments). **(C)** Intracellular production of IL-17 and IFNγ cytokine production by CD4+ T cells was analyzed by flow cytometry (CTL n = 5; HSD n = 5). **(D)** Treg presence was assessed by flow cytometry based on CD4+CD25+FOXP3+ expression (CTL n = 8; HSD n = 9; 2 independent experiments). Mann-Whitney test was used to compare the data between the 2 diets. Values are presented as means ± SD. “ns” not significant.

To investigate the HSD-linked difference in terms of disease severity between the two EAU groups, we first characterized and compared the IRBP-specific proliferation rate of autoreactive T cells and their cytokine production during the induction phase of the disease, on day 12 after immunization. Data are shown in **Figure 3**.

Overall, flow cytometry analysis shows a markedly increased proliferation rate of semi-purified CD3+CD4+ T cells in response to IRBP peptide stimulation, but no significant difference between HSD or CTL-diet fed mice (p = 0,8857) (**Figure 3A**).

Furthermore, specific ELISA tests were performed on the same cell culture supernatants to characterize the secretion of IL-17A, IFNγ and GM-CSF. As shown in **Figure 3B**, IRBP peptide stimulation shows a clear increase in cytokine secretion. However, there is no significant difference in terms of IL-17A (p = 0,3429), IFNγ (p = 0,6857) and GM-CSF (p = 0,2) cytokine secretion between mice fed with HSD compared to mice fed with CTL-diet. While IFNγ and GM-CSF secretion are rather stable between individual mice subjected to the same diet, IL-17A secretion fluctuates more (data not shown).

We also assessed at the intracytoplasmic level the cytokine production of CD4+ cells in mice subjected to CTL-diet versus HSD, confirming the results of the cytokine analysis of supernatants. Indeed, there are no statistically significant differences between diets in terms of IL-17A (p=0,8889) and IFNγ (p = 0,5476) production by IRBP peptide activated CD4+ T cells (**Figure 3C**).

As depicted in **Figure 3B**, under our experimental conditions HSD does not have any modulating effect on IL-17A secretion. However, this was not due to a default in intrinsic T cell capacities. Indeed, we checked, *in vitro*, 12 days after disease induction, the ability of CD3+CD4+ semi-purified T cells isolated from regular diet fed mice to proliferate and produce cytokines in response to a 48 hours’ stimulation by IRBP peptide, with or without addition of NaCl 40 mM to cell culture medium. The IRBP peptide *in vitro* stimulation induced a notable proliferation of CD3+CD4+ semi-purified T cells although with some variability between individual mice (data not shown) and the addition of NaCl to culture medium did not affect T cell proliferation with statistical significance (p = 0,4375). Regarding the cytokine secretion, IRBP peptide stimulation similarly induced IL-17A, IFNγ and GM-CSF production. NaCl addition to culture medium resulted in a statistically significant higher production of IL-17A compared to control medium (p = 0,0039) but did not affect IFNγ (p > 0,9999) nor GM-CSF (p = 0,0742) secretion (**Supplementary Figure S1**).

Lastly, we tested the effect of HSD on the presence of regulatory T cells (Treg) within autoreactive T cells, on day 12 after immunization. Treg percentages were assessed by flow cytometry based on CD4+CD25+FOXP3+ expression, comparatively between diets. Raw data from **Figure 3D** show that HSD does not affect Treg representation in diseased mice during the afferent phase (p = 0,9626). However, when calculating the respective ratios (IRBP/medium) of Tregs between control diet and HSD, there could be a trend towards a higher HSD-linked proportion of Treg cells following IRBP peptide stimulation (59% Treg in CTL diet versus 73% in HSD, data not shown).

### 3.3 Study of the integrity of the blood-retinal barrier by immunofluorescence

We investigated the effect of HSD on the BRB integrity by testing the expression of GFAP whose pattern expression is classically changed during retinal inflammation (27).

**Figure 4:**
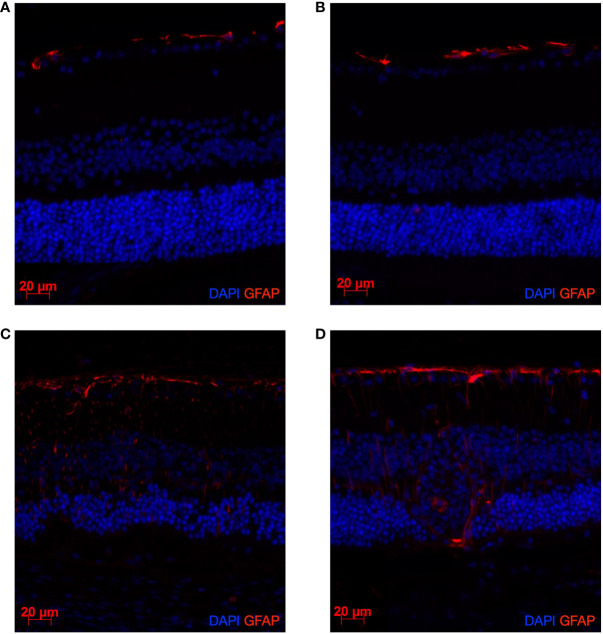
EAU induction leads to a clear activation of GFAP and alteration of the retinal structure. HSD does not affect GFAP expression nor retinal structure in both naïve and diseased mice. Naïve mice were sacrificed after 4 weeks of control diet or HSD. Diseased mice were similarly subjected to 4 weeks of control or HSD before being immunized and sacrificed 3 weeks after disease induction. Their eyes were collected and prepared for paraffin sectioning. Eye sections were stained for GFAP (red) detection. Cell nuclei were stained with DAPI (blue). GFAP expression in: **(A)** Naïve control diet-fed mice. **(B)** Naïve HSD-fed mice. **(C)** Native EAU control diet-fed mice. **(D)** Native EAU in HSD-fed mice.

As illustrated in **Figure 4**, by comparison with CTL-diet, HSD does not affect the BRB nor the structure of the retinal layers in both non immunized (**Figures 4A, B**) and immunized mice (**Figures 4C, D**). However, disease induction results in activation of GFAP and breakdown of the normal structure of the outer retinal layers in both diets (**Figures 4C, D**). Control stainings were performed and did not show any signal (**Supplementary Figure S2**).

### 3.4 Gut microbiome analysis on cecal samples

Overall, our previous data did not establish yet a clear link between the HSD effect on the disease-induced specific immune responses and the clinical phenotypes. Since gut microbiota has been highlighted as a key factor involved in inflammation and several pathologies related to inflammation, we evaluated the effects of HSD on the gut microbiota composition in naïve and diseased mice.

Gut microbiome analysis was performed on metagenomic DNA extracted from cecal samples. A taxonomic assignment of ASVs was performed and the samples were submitted for biostatistical analysis of α-diversity (metric measuring the microbiome diversity applicable to a single sample) and β-diversity (metric measuring the similarity or dissimilarity between two communities). Due to experimental constraints, non-immunized, native EAU and EAU by AT mice cannot be compared directly. The effect of HSD on gut microbiome was thus analyzed by comparison with control diet, within each cohort, separately.

#### 3.4.1 Effect of HSD on α- and β-diversity

**Figure 5:**
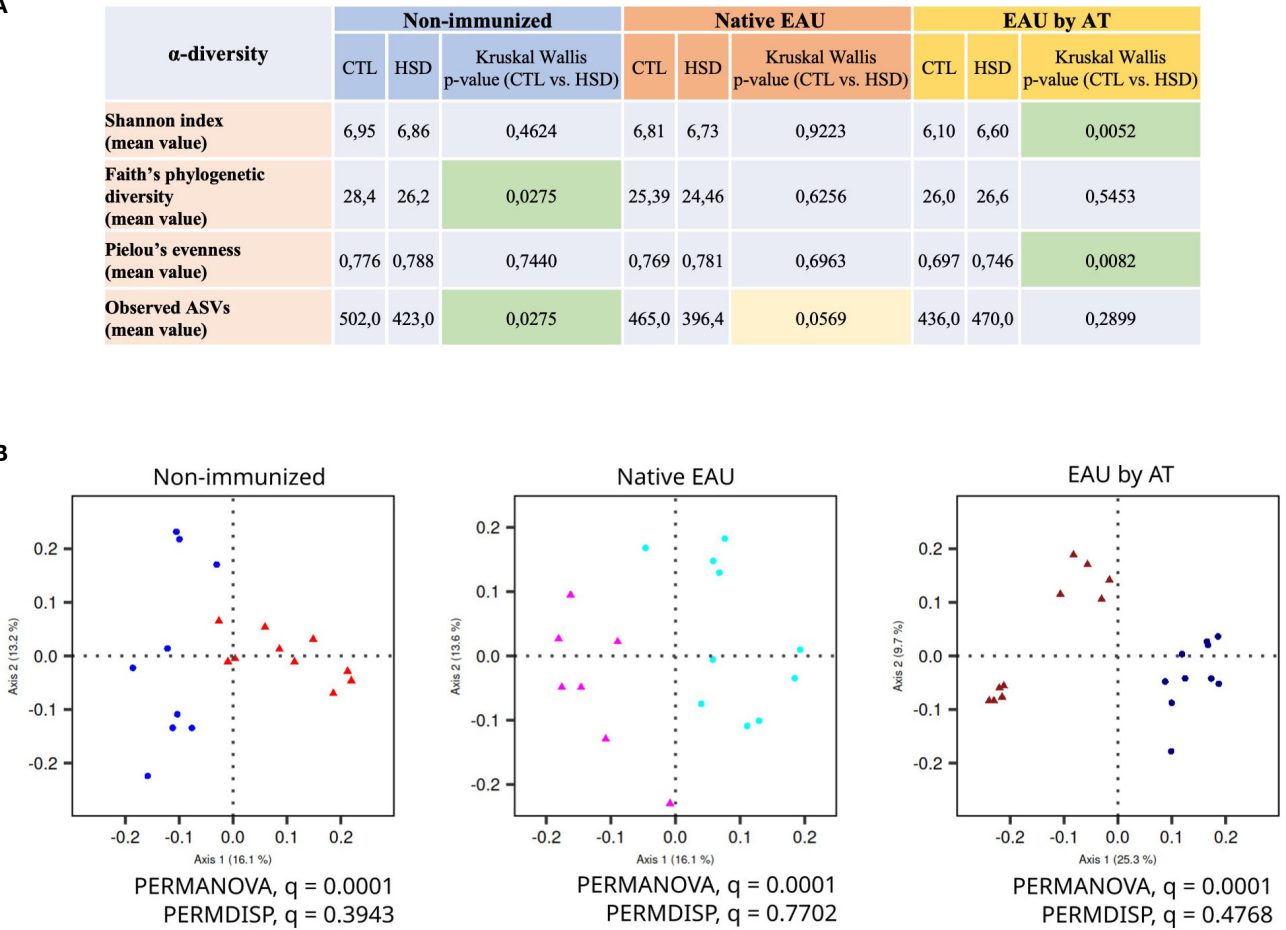
HSD modifies gut microbiota composition in healthy and diseased mice. Due to experimental constraints, the effect of HSD on gut microbiota was analyzed on cecal samples within each cohort separately. HSD affected gut microbiota composition in terms of **(A)** α-diversity metrics (measure of the microbiome diversity applicable to a single sample) and **(B)** β-diversity metrics (measure of similarity or dissimilarity between two communities) illustrated by Unweighted UniFrac PCoA. In the panel 5B, the control groups are depicted with dots and the HSD groups with triangles.

Firstly, our findings demonstrate that the α-diversity metrics are differently affected by exposure to HSD (**Figure 5A**). In non-immunized mice, HSD decreased the gut microbiota Faith’s phylogenetic diversity and the number of observed ASVs (richness) with statistical significance but did not affect Shannon index and Pielou’s evenness. This suggests that HSD reduced the number of different types of bacteria in the gut without affecting the distribution of their relative abundances. In diseased mice, we observed a difference in terms of α-diversity between the two EAU groups. In native EAU mice, our data showed no significant difference between diets in terms of α-diversity, although the number of observed ASVs tended to be lower in mice fed with HSD. In EAU by AT mice, HSD increased the gut microbiota Shannon index and Pielou’s evenness with statistical significance but did not affect Faith’s phylogenetic diversity and observed ASVs. This suggests that in this EAU by AT model the HSD promoted a more even distribution between the different types of bacteria.

Secondly, **Figure 5B** shows the β-diversity metrics illustrated by Unweighted UniFrac PCoA (Principal Component Analysis). The data clearly indicate that the HSD significantly affected gut microbiota composition in control condition (non-immunized mice) and in autoimmune uveitis (native EAU and EAU by AT). Indeed, in all groups, the PERMANOVA q-values are significant (= 0,0001) while the PERMDISP q-values are not.

#### 3.4.2 Family- and genus-level global taxonomic analysis

**Figure 6:**
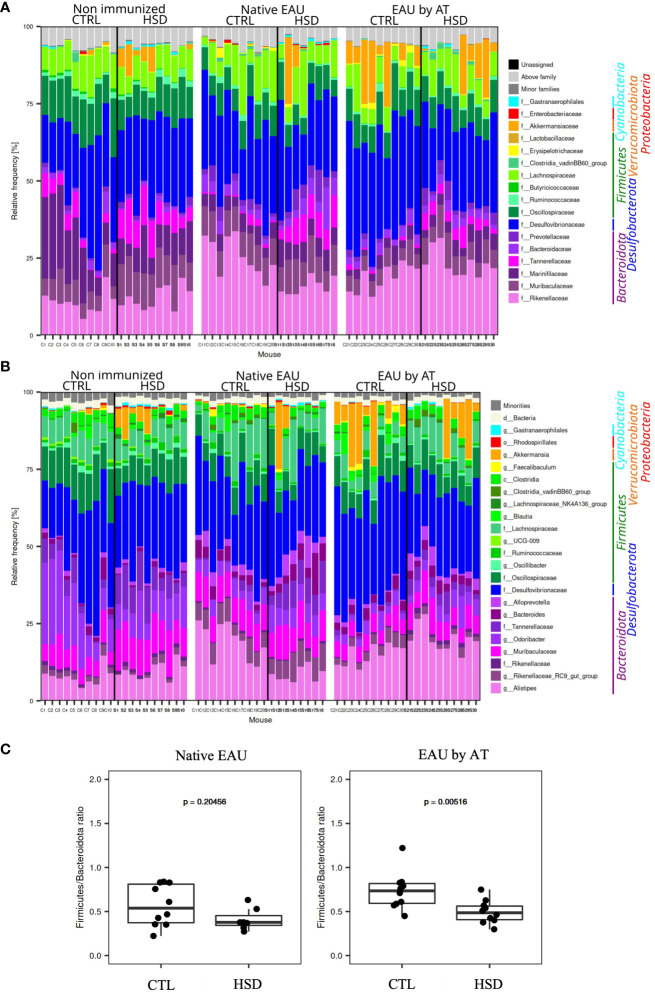
Analysis of the mouse gut microbiota showed significant differences between mice fed with the control (CTL) or high-salt diet (HSD). Taxonomy barplots for each sample from non-immunized mice, mice with native EAU, and mice with EAU by AT, fed either with CTL or HSD: **(A)** family- and **(B)** genus-level. Taxa are ordered according to the mean relative frequency on phylum level and grouped in phyla by color. **(C)** *Firmicutes/Bacteroidota* ratio in native EAU and EAU by AT.

To go deeper in the analyses and identified bacterial taxa affected by HSD, we analyzed the relative abundances of the different ASVs (amplicon sequence variants) in the samples. ASVs are defined as unique DNA sequences present in the samples and are the most detailed view of the microbiota we could obtain with the methodology used in this study. A taxonomic assignment was first performed to determine what bacterial genus each ASV belongs to. For some ASVs, we could not determine the genus, so we assigned to a higher taxonomic level (family, order…). Data are shown in **Figure 6**.

When analyzing more precisely, at a family- and genus-level, the gut microbiota in healthy and diseased mice, the taxonomy barplots showed that in general *Bacteroidota* (*Alistipes*, *Rikenellaceae RC9 gut group*, *Odoribacter*, *Bacteroides*…), *Desulfobacterota* (*Desulfovibrionaceae*), and *Firmicutes* (*Oscillobacter*, *Lachnospiraceae*,…) were the main phyla in all samples and that *Verrucomicrobiota* (*Akkermansiaceae*) were well represented in some samples (**Figures 6A, B**).

As concerns the effect of HSD on the *Firmicutes/Bacteroidota* (F/B) ratio whose increase was frequently associated to pro-inflammatory properties in autoimmune diseases, our data in **Figure 6C** show that in native EAU mice there was no statistically significant difference in terms of F/B ratio depending on HSD. On the contrary, EAU by AT mice fed with HSD had a significantly lower F/B ratio.

#### 3.4.3 Effect of HSD on the abundance of ASVs and taxa

The further identification of the respective bacterial taxa and ASVs significantly differentially abundant between mice subjected to CTL diet versus HSD was done separately for each group (non-immunized, native EAU and EAU by AT).

Data from **Table 1** show that unknown *Tannerellaceae* was the only taxon associated to exposure to HSD in all treatment groups. Besides, interestingly, unknown *Gastranaerophilales* were significantly more abundant after HSD in the native EAU group, only.

**Table 1:** Respective bacterial taxa and ASVs detected with statistically significant difference in cecal samples of healthy and diseased mice subjected to HSD or CTL diet.

		Non-immunized	Native EAU	EAU by AT
**Taxa**	HSD > CTL	*Lachnospiraceae* NK4A136 group Unknown *Tannerellaceae*	Unknown *Gastranaerophilales* Unknown *Tannerellaceae*	*Alistipes* *Alloprevotella* Unknown *Bacteroidales* Unknown *Bacteria* *Clostridia* vadinBB60 group Unknown *Lachnospiraceae* Unknown *Muribaculaceae* *Odoribacter* Unknown *Ruminococcaceae* Unknown *Rikenellaceae* Unknown *Tannerellaceae*
	CTL > HSD	/	/	*Blautia* *Faecalibaculum* Unknown *Lachnospirales*
**ASVs**	HSD > CTL	*Alistipes* Unknown *Desulfovibrionaceae* Unknown *Muribaculaceae* (2) Unknown *Rikenellaceae* Unknown *Tannerellaceae* (3)	*Alistipes* Unknown *Bacteria* Unknown *Desulfovibrinaceae* Unknown *Gastranaerophilales* (2) Unknown *Muribaculaceae* (2) *Odoribacter* (2) *Rikenellaceae* RC9 gut group Unknown *Tannerellaceae*	*Alistipes* *Alloprevotella* Unknown *Bacteria* *Bacteroides* Unknown *Bacteroidales* *Clostridia* vadinBB60 group Unknown *Desulfovibrionaceae* Unknown *Lachnospiraceae* Unknown *Muribaculaceae* *Odoribacter* Unknown *Rikenellaceae* Unknown *Tannerellaceae*
	CTL > HSD	Unknown *Oscillospiraceae* (2)	*Alistipes* (3) Unknown *Oscillospiraceae* Uncultured *Ruminococcaceae*	*Akkermansia* *Alistipes* Unknown *Bacteria* *Bacteroides* *Blautia* Unknown *Desulfovibrionaceae* *Faecalibaculum* Unknown *Lachnospiraceae* Unknown *Lachnospirales* Unknown *Muribaculaceae* *Odoribacter* Unknown *Oscillospiraceae* *Rikenellaceae* RC9 gut group *Tuzzerella*

/ = none.

In addition, more differentially abundant ASVs and taxa were present in the EAU by AT group, whatever the diet. In several taxa, some ASVs were more abundant in CTL-fed mice and others in HSD-fed mice, indicating intra-taxon diversity in response to HSD or its downstream effects.

## 4 Discussion

Western diet, and more specifically high salt intake, is mainly associated with cardiovascular diseases. Recent studies also reported a link between high salt diet (HSD) and clinical exacerbation in various mouse models of autoimmune diseases, mainly through increased differentiation of T cells into highly pathogenic Th17 cells (28). In addition, many studies have demonstrated HSD-induced dysbiosis and few studies suggested a link between dysbiosis induction and disease exacerbation (16, 29). However, the effect of HSD on autoimmune diseases remains controversial and not fully understood.

In this study, our aim was to investigate the effects of HSD on the development of experimental autoimmune uveitis (EAU) in mice. Different parameters were examined comparatively between healthy and diseased mice, subjected to HSD versus control diet. Unexpectedly, our data first showed a significant attenuating effect of HSD on the clinical severity of the induced native EAU but no effect on EAU by adoptive transfer (AT). In order to investigate which mechanisms supported such difference in disease development, we first characterized ex vivo the IRBP antigen-specific response of T cells isolated from IRBP-immunized animals under control diet or HSD during the induction phase of the disease (day 12). Overall, we did not observe significant differences between diets in terms of T helper cell proliferation rate nor their cytokine production. There was perhaps a trend towards a higher HSD-linked proportion of regulatory T cells (Treg), but not statistically significant. As a whole, those data showing no HSD-linked alterations of autoreactive T cells during the induction phase of the disease cannot explain the attenuating effect of HSD on the severity of native EAU, but they are in agreement with the absence of effect of HSD on the severity of EAU induced by AT of semi-purified autoreactive T cells. Since gut microbiota has already been highlighted to be involved in inflammation and in several autoimmune diseases (30), we also decided to assess the effect of HSD on the gut microbiota composition in naïve and diseased mice. Our analysis of the intestinal microbiota on cecal samples showed that an exposition to HSD affected the gut microbiota composition in each separate group. However, in our study, the effects of HSD on the gut microbiota also depended on what disease model was considered, suggesting that immunization protocol, diet duration or, less likely, mouse age could play a role in the gut microbiota composition as well, potentially impacting phenotypic characteristics of the mice. In accordance, many factors were reported by different research groups to drive the gut microbiota composition, including the different mouse providers (31), the housing conditions and sanitary status of the animal facility or by the analysis of fecal versus cecal samples (32).

HSD is classically associated with worsening of autoimmune diseases. Indeed, an exacerbating effect of dietary sodium has been demonstrated in diverse murine models of autoimmune diseases (13), among which inflammatory colitis (32, 33), rheumatoid arthritis (34) and lupus nephropathy (35). Similarly, Kleinewietfeld et al. (12) showed a deleterious effect of HSD on the EAE induced in C57BL/6 mice. Our data showing an attenuating effect of HSD on native EAU are therefore somewhat surprising. However, in accordance with our work, Na et al. (36) showed a protective role of HSD in a spontaneous multiple sclerosis model. They further did not reproduce the disease worsening effect of HSD in EAE C57BL/6 mice. Martín-Hersog et al. (37) even showed an improvement in clinical status after 51 days of NaCl administration through a nasogastric tube in EAE Agouti rats. The detrimental effect of salt was also not detected in mouse models of spontaneous and antigen-induced experimental autoimmune thyroiditis (38), nor by Krementsov et al. (28) in an EAE model in SLJ/JCrHsd male mice.

Diverse biological mechanisms have been investigated by several research groups to explain the worsening or protecting effect of HSD. As concerns a potential effect of HSD on health conditions, we never observed any obvious phenotypic difference between mice subjected to control or HSD. Besides, we did measure the osmolality of blood serum harvested from the retro-orbital sinus in each treatment group, which did not show any significant difference in response to exposure to HSD (data shown in **Supplementary Figure S3**). Literature also showed that HSD at same salt concentration had no impact on blood pressure (12, 39), nor on body weight (40). On the other hand, Kleinewietfeld et al. (12) highlighted an increased differentiation of Th17 cells as linked to a worsened clinical outcome in EAE-induced mice after exposure to HSD. However, this effect of HSD was not reproduced by other groups (28, 36). In accordance, we did not observe any difference in specific autoimmune systemic reaction in our EAU model. Apart from the role of Th17 cells, other mechanisms have been related to exposure to HSD. Na et al. (36) suggested that a higher level of circulating corticosterone could increase the expression of tight junction molecules and result in an enhanced tightening of the blood-brain barrier. They hypothesized that high salt concentrations could suppress the renin-angiotensin-aldosterone system (RAAS) and thereby attenuate inflammation. In addition, another research group suggested that sustained high salt intake induces glucocorticoid excess through activation of the hypothalamic-pituitary-adrenal axis (41). On the contrary, Krementsov et al. found that HSD did result in increased blood-brain barrier permeability (28). However, in our work, if EAU induction led to a clear activation of GFAP expression and alteration of the retinal structure, HSD did not affect GFAP expression nor retinal structure in both naïve and diseased mice.

Another important mechanism to explain how HSD modulates autoimmune diseases is through gut microbiota modification. Indeed, several research groups reported that gut microbiota composition modulates the severity of autoimmune diseases like EAU (14, 42), EAE (12, 40, 43) and colitis (29, 33). Moreover, Dusek et al. (44) showed that probiotic administration of live *Escherichia coli* Nissle 1917 controls inflammation and clinical severity in EAU and seems to participate in modulating the so-called “gut-retina axis”. Even though some specific modifications of the gut microbiome have been described in recent years, those studies targeted either exposure to HSD or disease induction, but rarely associated both. This made the originality of our study.

Regarding our microbiome study, we analyzed the gut microbiota separately between each group (non-immunized, native EAU and EAU by AT) in order to avoid any experimental bias. Indeed, many variables could be linked to the different disease induction protocols between native EAU and EAU by AT [see experimental schedule (**Figure 1**)]. Our findings first demonstrated that α-diversity (measure of the microbiota diversity in one sample) was differently affected between groups by exposure to HSD, whereas β-diversity (measure of between-sample differences in microbiota diversity) was significantly modified by HSD in all groups. We found that HSD reduced the gut microbiota richness in non-immunized mice. Our observations were consistent with the literature as Hamad et al. (45) showed a lower richness and Faith’s diversity in naïve mice fed with 4% NaCl and distinct clusters on the PCoA plot of the gut microbiota composition depending on the sodium content in the food.

As concerns the identification of the bacterial taxa and ASVs significantly differentially abundant between mice subjected to control diet versus HSD, our data globally showed that unknown *Tannerellaceae* was the only taxon associated to exposure to HSD in all treatment groups. Comparison of its major ASV, which was also consistently differentially abundant in different treatment groups, to the NCBI databases showed perfect matches to *Parabacteroides goldsteinii* and imperfect matches to other *Parabacteroides* species, so this ASV most likely represented *P. goldsteinii* or a related *Parabacteroides* species.

Secondly, interestingly, we identified a significantly higher abundance of unknown *Gastranaerophilales* in HSD-fed native EAU mice, only. This might be of interest as we observed beneficial effects of HSD on clinical grades specifically in native EAU mice. This hypothetical link between *Gastranaerophilales* and clinical outcome of EAU is totally new since there is no known prior literature linking *Gastranaerophilales* and uveitis. *Gastranaerophilales* are known to be adapted to anaerobic life in the gut of host organisms (46, 47) and based on predictions from genomic data, they are thought to benefit their host by producing vitamins K1 and K2 and aiding digestion (47). Furthermore, genome-based metabolic modeling indicated that at least some *Gastranaerophilales* species produce indole. Indole and its derivatives from tryptophan metabolism, such as indole propionic acid, may pass into the bloodstream, as observed in a study of Parkinson’s disease patients (48). Indole propionic acid is also known to exert anti-inflammatory effects (49–51). Such properties of *Gastranaerophilales* could be linked to the decreased clinical severity of native EAU under HSD. Accordingly, *Gastranaerophilales* has recently been shown to be increased in improved DSS-induced acute colitis mice.

Lastly, we found that HSD seems to affect more ASVs and taxa in AT than in native EAU. Such larger number of ASVs and taxa differentially abundant in the EAU by AT group, despite different diets and similar clinical grades, could have occurred due to a longer exposure to HSD and/or due to a different disease induction protocol.

In naïve mice, Wang et al. (52) showed that exposure to HSD increased *Firmicutes*/*Bacteroidota* (formerly *Bacteroidetes*) ratio and the abundances of *Lachnospiraceae* and *Ruminococcus* but decreased the abundance of *Lactobacillus*. Hamad et al. (45), Wilck et al. (16), Miranda et al. (29) and Ferguson et al. (53) described a significant enrichment of *Akkermansia*, *Alistipes* and *Clostridia* and a depletion of lactic acid-producing bacteria in a dose-dependent manner. In the context of experimental autoimmune diseases, a “pro-inflammatory profile” can be proposed. An increased *Firmicutes/Bacteroidota* ratio, a decreased abundance of *Lactobacillus*, *Akkermansia muciniphila* and protective short-chain fatty acid production are recurrent modifications of the gut microbiota associated with exacerbated disease (29, 40, 47–49, 54–56). Regarding our uveitis models, such specific pro-inflammatory modifications were not observed (42). Accordingly, within our experimental model combining HSD and EAU, we did not find a significant HSD-linked decrease in *Firmicutes*/*Bacteroidota* ratio which could be correlated to the lower severity of the native EAU.

In conclusion, our data show a differential effect of HSD on the induction of EAU, by decreasing the severity of native EAU but not modulating the EAU by AT. Accordingly, exposure to HSD does not affect the afferent retinal antigen specific immune responses nor the BRB integrity in diseased mice. However, our study suggests a possible impact of salt diet on the gut microbiota composition and consequently on the development of EAU, potentially linked to the presence of specific bacterial families: unknown *Tannerellaceae* for exposure to HSD and unknown *Gastranaerophilales* for HSD-reduced severity of native EAU. Further studies are however required to investigate the potential beneficial role of *Gastranaerophilales* in native EAU induction.

## Data availability statement

The datasets presented in this study can be found in online repositories. The names of the repository/repositories and accession number(s) can be found below: https://www.ebi.ac.uk/ena/browser/view/PRJEB65938, PRJEB65938.

## Ethics statement

The animal study was approved by Comission d’Ethique du Bien-Être Animal, CEBEA, Faculté de Médecine, Université Libre de Bruxelles. The study was conducted in accordance with the local legislation and institutional requirements.

## Author contributions

ND: Data curation, Formal analysis, Funding acquisition, Investigation, Writing – original draft, Writing – review & editing, Project administration. VFo: Methodology, Writing – review & editing, Investigation. MV: Writing – review & editing, Data curation, Formal analysis, Software, Validation, Writing – original draft. RA: Investigation, Writing – review & editing. LV: Writing – review & editing, Investigation, Funding acquisition. DL: Investigation, Writing – review & editing, Methodology. VFl: Investigation, Methodology, Writing – review & editing, Supervision. AE: Methodology, Writing – review & editing, Supervision, Formal analysis, Validation. CB: Conceptualization, Data curation, Formal analysis, Investigation, Methodology, Project administration, Supervision, Writing – original draft, Writing – review & editing, Validation. FW: Formal analysis, Supervision, Validation, Writing – review & editing, Conceptualization, Funding acquisition, Resources.
