# Supplementary material for: High salt diet alleviates disease severity in native experimental autoimmune uveitis
Source: Front Ophthalmol (Lausanne). 2024 May 31;4:1370374. doi: 10.3389/fopht.2024.1370374 (PMC11182228; doi:10.3389/fopht.2024.1370374)
Supplement: Supplementary file 1 [file DataSheet_1.docx]

Supplementary Material


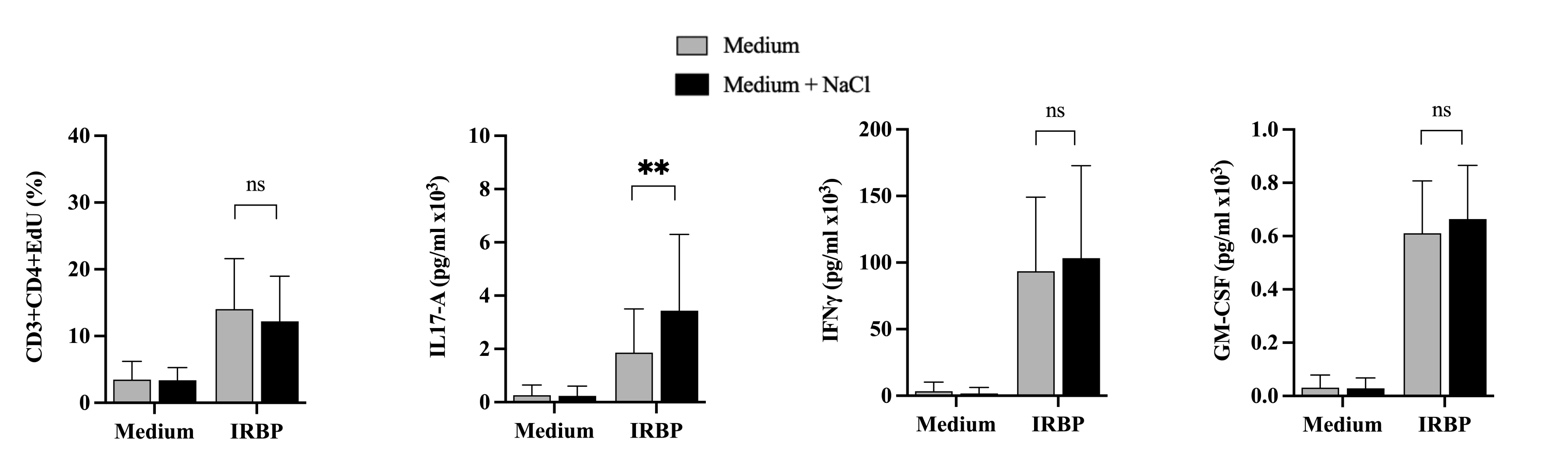


**Supplementary Figure S1.** **IRBP peptide-specific T cells secrete significantly more IL-17A after in vitro exposure to 40mM NaCl.**Twelve days after s.c. immunization with IRBP peptide emulsified in CFA and combined with an i.p. injection of PTX, regular diet fed mice were sacrificed and their spleen and draining lymph nodes collected and dissociated. Semi-purified T cells were cultured with or without addition of 40mM NaCl in culture medium.

Wilcoxon test was used to compare the effect of in vitro NaCl addition.
Values are presented as means ± SD, n = 9 mice/group.
ns not significant; ** p < 0,01

**
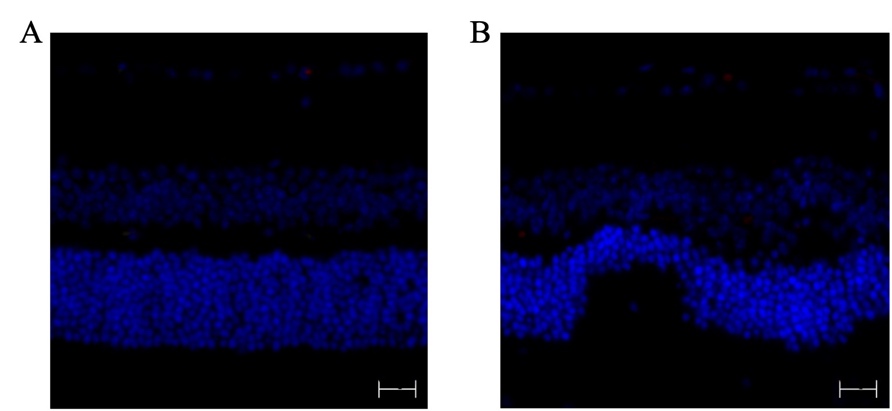
**

**Supplementary Figure S2. Negative control for GFAP staining on eye sections.**Paraffin-embedded eye sections were stained by omitting anti-GFAP primary antibody.
**(A)** Non-immunized mice.
**(B)** Native EAU mice.
The scale indicates 20 µm.


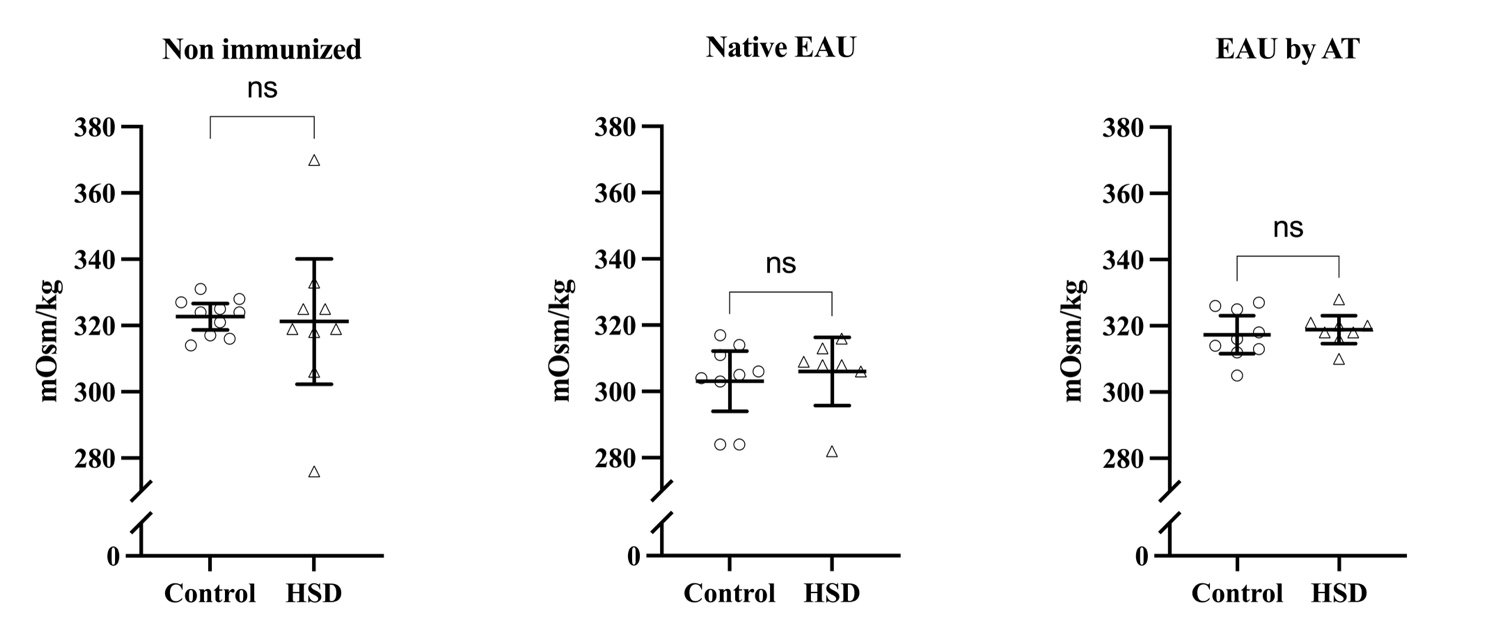


**Supplementary Figure S3 : Serum osmolality measured in blood harvested from the retro-orbital sinus was not influenced by exposure to HSD.**
Serum osmolality harvested from the retro-orbital sinus was compared between mice on control diet versus HSD among non-immunized (n=10 and n=9 respectively), native EAU (n=9 and n=7 respectively) and EAU by AT (n=9 and n=8 respectively) mice. The data showed no statistically significant difference in serum osmolality between the control diet and HSD, either in non-immunized (p=0.8263), native EAU (p=0.4854) or EAU by AT (p=0.4935) mice.

The osmolality was measured with a cryoscopic osmometer (OSMOMAT^®^ 030 – Gonotec) following manufacturer’s instructions.

Mann-Whitney test was used to compare serum osmolality between the two diets.
Values are presented as means ± 95% CI.
ns not significant
